# Associations between Atopic Dermatitis and Behavior Difficulties in Children

**DOI:** 10.3390/medicina60030492

**Published:** 2024-03-17

**Authors:** Inga Kisieliene, Beatrice Aukstuolyte, Antanas Mainelis, Odilija Rudzevicienė, Matilda Bylaite-Bucinskiene, Andreas Wolenberg

**Affiliations:** 1 Clinic of Infectious Diseases and Dermatovenereology, Vilnius University, 03225 Vilnius, Lithuania; 2 Dr Jonas Basanavičius Military Medical Service, 66102 Druskininkai, Lithuania; 3 Faculty of Mathematics and Informatics, Vilnius University, 03225 Vilnius, Lithuania; 4 Clinic of Children’s Diseases, Faculty of Medicine, Vilnius University, 03225 Vilnius, Lithuania; 5 Department of Dermatology and Allergy, Augsburg University Hospital, 86156 Augsburg, Germany; 6 Department of Dermatology and Allergy, Ludwig-Maximilian University of Munich, 82152 Munich, Germany; 7 Comprehensive Center for Inflammation Medicine, University of Luebeck, 23562 Luebeck, Germany

**Keywords:** atopic eczema, atopic dermatitis, behavioral difficulties, child behavior checklist, pediatric

## Abstract

*Background and Objectives*: There has been increasing evidence that atopic dermatitis (AD) is associated with behavioral difficulties (BDs). There is currently a lack of evidence of how the severity of the disease determines BDs and what additional factors may contribute to their manifestation. The aim is to determine what kind of BDs occur in children with AD compared to healthy children and to find out what additional factors may contribute to the development of BDs in AD patients. Materials and Methods: This is a cross-sectional, prospective study with the application of a risk assessment instrument for behavior difficulties (Child Behavior Checklist, CBCL 6/18) in pediatric patients with AD and healthy controls (6–17 years) between 1 January 2020 and 31 December 2022. For statistical comparison, mainly Wilcoxon–Mann–Whitney and Student’s *t*-test were used, considering a significance level of 5%. *Results*: This study included a total of 101 children: 48% with AD, 52% non-AD. The mean age was 10 ± 2.7 years for AD, and10.5 ± 3.1 years for the control patients. AD patients had higher internal behavior scale scores and T-scores (6.6 ± 6.4 vs. 9.6 ± 6.9 and 47.9 ± 9.5 vs. 52.3 ± 10.2, *p* = 0.01), anxiety/depression scale score and T-score (2.8 ± 2.7 vs. 4.3 ± 3.5 and 47.7 ± 8.4 vs. 52.5 ± 11, *p* = 0.02), and somatic problems scale score and T-score (2.1 ± 2.3 vs. 3.5 ± 3 and 47.6 ± 8.5 vs. 52.7 ± 10.9, *p* = 0.005). Patients with severe AD had sleep disturbance and itching scores higher than those with mild–moderate AD (5.4 ± 2.6 vs. 2.4 ± 2.2, *p* = 0.000 and 6.6 ± 2.4 vs. 4 ± 2.8, *p* = 0.001). The mean morning serum cortisol concentration was lower in AD patients compared to controls (252.91 ± 304.34 vs. 351.55 ± 126.09 nmol/L, *p* = 0.047). *Conclusions*: Children with AD present a higher risk of BDs than healthy controls. Patients with severe AD experience more sleep disturbances and a greater intensity of itching compared to mild–moderate AD. The occurrence of BDs was not related to serum cortisol levels. The cortisol level, severity, age, gender, duration of illness, intensity of pruritus, and sleep disturbance did not affect the development of BDs.

## 1. Introduction

Atopic dermatitis (AD) is a chronic, pruritic inflammatory skin disease that usually develops in infancy and affects 10 to 20% of children and 1 to 3% of adults. It is characterized by localized or widespread itchy skin lesions, with a relapsing course ranging from mild-to-severe clinical form [1,2,3]. The symptoms of AD affect the psychological and mental health of patients, mainly due to the chronic relapsing disease course, intense pruritus, unaesthetic appearance of lesions, and sleep disturbances. AD is the most consistent, chronic, and severe of the pruritic disorders. AD is characterized by itching, which is often strongest at night and causes scratching, which can interfere with falling asleep and sleep disruptions [4,5]. The itch of eczema is thought to be multi-factorial, with impaired temperature regulation, inflammatory cytokines, epidermal barrier function, sensory sensitivity, and skin dryness, all causally implicated. In particular, vasodilatation and erythema impact the itch of eczema and are associated with both increased and reduced skin temperature [6]. Sleep and circadian researchers have extensively documented the nightly vasodilation and warming of the skin that promotes heat loss necessary for the nightly lowering of the core body temperature. The nightly elevation in skin temperature is associated with trans-epidermal water loss and reduced epidermal barrier function which facilitates the entry of irritants and itch-causing agents [5]. Corticosteroid levels are highest in the evening, so the threshold for inflammatory reactions may be lowered during the night. Variations in corticosteroid levels have been suggested to play a role in the nighttime exacerbation of asthma, so it would not be surprising to find an association with certain kinds of itching caused by inflammation [5]. Adequate sleep is essential for children’s well-being and health, and sleep disturbances have been linked to a wide range of cognitive, mood, and behavioral problems, as well as poor educational performance [7,8]. Sleep disturbances have been identified as a major cause of reduced quality of life in atopic dermatitis [4,9]. The interference of itching with sleep may place patients at risk for behavioral and neurocognitive deficits [4].

The mental health of AD patients has already been investigated, and it has been found that the disease may have contributed to the development of mental and behavioral disorders [10,11,12,13,14]. A meta-analysis of 16 cross-sectional studies found that AD in adults was positively associated with higher scores on a depression scale, parental depression, the use of antidepressants, and suicide, and the association was stronger in those with higher disease severity [13]. In a cross-sectional prospective population-based cohort study by Zhang et al., AD was shown to have positive associations with chronic fatigue syndrome, burnout, depression, social phobia, panic disorder, attention-deficit/hyperactivity disorder, and eating disorders [14]. Mental difficulties can impair the response to treatment and the physical illness itself, which in turn can have a negative impact on mental health and quality of life [10]. There is a growing number of studies on quality of life, sleep, and psychiatric comorbidities in AD, but limited knowledge and research are assessing how AD is associated with mental disorders in children and how these are related to the severity of the disease [10,15,16,17]. The neural mediation between stress and AD exacerbations varies for each end. Some specific areas of the peripheral and central nervous system are physiologically activated by stressful stimuli. The hypothalamic–pituitary–adrenal (HPA) axis and the autonomic nervous system are stimulated during stress. They are closely linked to the immune system and are involved in the pathogenesis of stress-induced diseases. Markers of this reaction can be found in saliva or serum, but there are no reliable biomarkers to assess the stress levels of AD patients [18]. Since the hypothalamic–pituitary–adrenal (HPA) axis is known to help regulate inflammation through the regulation of cortisol, investigators have examined the role of the HPA axis and endogenous cortisol in the outcome of allergic disorders [19]. Cortisol is a hormone produced in the adrenal cortex, with intense synthesis in the second half of the night and peak serum levels between 7 and 8 am. During the day, cortisol levels drop, and at night, only about 10% of the morning cortisol remains in the body. Cortisol synthesis is facilitated by physical or psychological stress. This is followed by the activation of the metabolism, an increase in the body’s energy supply, and changes in the activity of other hormones. Persistent stress can lead to persistently elevated levels of cortisol in the body. It has been hypothesized that after prolonged physical and/or mental stress, the HPA axis is affected and becomes less sensitive, resulting in a decrease in cortisol production in the adrenal glands [20].

Our study aims to assess the prevalence and pattern of behavioral difficulties in children with AD and to determine the associations with the severity or duration of the disease, pruritus, and sleep disturbance. We also investigated patients’ serum morning basal cortisol levels and looked for an association with behavioral difficulties.

## 2. Materials and Methods

This cross-sectional prospective study was conducted at Vilnius University Hospital Santaros Klinikos Clinic of Children’s Diseases, between December 2020 and December 2022. Children with AD and healthy children from 6 to 17 years, who came to a dermatovenereologist consultation, were invited to participate in the study. Patients were recruited by consecutive sampling based on their clinic appointment. Eligibility criteria for the AD group were defined as individuals living in Lithuania, who were clinically diagnosed with AD according to Hanifin and Rajka’s diagnostic criteria for AD. The control group was composed of patients with no history of eczema, who presented for a general skin examination. The exclusion criteria included any severe exacerbated chronic, congenital, or oncological disorders that could affect the mental health of patients. Those who were not able to understand the questionnaire, were illiterate, or declined participation were excluded. All participants agreed to participate in this study and signed the consent form (the consent form was signed by both parents or caregivers and by children older than 12 years). This study was approved by the Vilnius Regional Biomedical Ethics Committee (No. 2020/8-1251-733).

All characteristics were obtained through patients and parental questionnaires. Participants had to fill out an original questionnaire, which included 45 questions, created by the authors, for demographic and epidemiological data. The format of the questionnaire included multiple-choice questions, yes/no questions, Likert scales, and open-ended questions.

Behavioral difficulties were evaluated using the 2001 Child Behavior Checklist for Ages 6–18 (CBCL 6/18), validated for the Lithuanian language [21]. Assessing the emotional and behavioral aspects in scientific studies is challenging because of the subjectivity of symptoms. In this context, using validated instruments allows for quantification and comparisons based on established cut-off points, making the evaluation more precise and standardized [17]. CBCL 6/18 is one of the most widely used instruments for screening psychological disorders. It is part of the Achenbach System of Empirically Based Assessment (ASEBA), validated by many scientific societies and cultural groups [17]. Parents answer a questionnaire consisting of 138 questions, distributed as follows: the first 20 questions relate to social competence, divided into participation in activities, social relationships, and school, while the remaining 118 questions relate to behavioral problems. The CBCL 6/18 assesses the presence and profile of behavioral disorders, which can be an internalizing profile with a tendency towards depression, anxiety, and somatic disorders or an externalizing profile with aggressive and rule-breaking behavior [17,21]. Questions are classified into eight syndromes: “Anxiety/Depression”, “Isolation”, and “Somatic Complaints”, grouped as internalizing problems; “Rule-Breaking Behaviour” and “Aggressive Behaviour”, grouped as externalizing problems. The syndromes “Social Problems”, “Thought Problems”, and “Attention Problems” are evaluated separately. The CBCL 6/18 consists of questions that should be answered as “absent/not true” (score = 0), “sometimes/slightly true” (score = 1), or “often true” (score = 2). At the end, it contains open-ended questions for parents. The sum of scores is converted into T-scores according to analyses that are appropriate for each sex and age. The T-score is based on a bell curve distribution, with a mean of 50 and a standard deviation of 10. A T-score below 64 is Normal; between 65 and 69, Borderline; and equal to or above 70, Clinical [16]. The CBCL 6/18 questionnaire was administered by a psychologist (co-author B.A.) licensed to evaluate the CBCL 6/18. The data from the questionnaires were entered into a specific software that generated T-scores and classified according to the Lithuanian validation. The classification of T-scores varies according to the analyzed scale, with the normal range being > 37 for total social competence, >31 for the scales analyzed separately (social, activity, and school), less than or equal to 69 for emotional and behavioral problems, and 63 for internalizing and externalizing problems [21].

The skin condition was evaluated by a dermatovenereologist (co-author, I.K.,), and the severity of AD was determined by using the SCORing AD (SCORAD) index [22]. The index is based on individual items about the intensity of the affected area, including erythema, edema/papulation, oozing/crusts, excoriations, lichenification, and dryness, with each item rated on a scale of 0 to 3. The extent of the affected area is determined using the ‘rule of nines’ with a maximum score of 83. The subjective symptoms are the effects of daytime pruritus and sleep loss rated on an analog scale of 0 to 10. The tool assesses disease severity with a maximum score of 103 and categorizes the severity as mild (<25), moderate (25–50), or severe (>50) [23]. Both the CBCL 6/18 and the SCORAD index are among the most widely used outcome measurements, allowing for comparisons of the results with other authors’ data.

All statistical analysis was performed using the R (v. 4.0.4) program package. The mean, standard deviation (SD), quartiles (Q1 and Q3), median, and the available number of observations of the quantitative variables are presented. Categorical variables are presented as the absolute amount and the percentage. To test hypotheses between the two groups’ comparison of the quantitative variables, Student’s *t*-Test or the nonparametric Mann–Whitney U test was used as appropriate. Normality was tested using the Shapiro–Wilk test. To test multiple variables related to the scores of the scales, multivariate linear regression was used. A *p*-value less than 0.05 was considered significant.

## 3. Results

This study included a total of 101 children: 48 (48%) children with AD and 53 (52%) non-AD patients. The mean age was 10 ± 2.7 median 9 (8–11) years for AD patients and 10.5 ± 3.1, median 11 (8–13) years for the control patients. The distribution of genders in the groups showed that 26 (57%) in the control group and 20 (43%) in the AD group were boys. Of the adults who completed the questionnaires, 93 (92%) were mothers and 8 (8%) were fathers. The demographic and clinical characteristics of the participants are presented in Table 1.

The mean SCORAD score for AD patients was 41.75 ± 20.95. The mean age, sex ratio, and family history of allergy did not differ between controls and patients with AD. Although the duration of disease was not different between the two AD groups (mild-to-moderate and severe), however, sleep disturbance and pruritus intensity points were higher in the severe AD group than in the mild-to-moderate AD group (2.4 ± 2.2 vs. 5.4 ± 2.6, *p* = 0.000 and 4 ± 2.8 vs. 6.6 ± 2.4, *p* = 0.001).

The mean morning serum cortisol concentration was lower in AD patients compared to controls (252.91 ± 304.34 vs. 351.55 ± 126.09 nmol/L) and there was a statistical difference between the two groups (*p* = 0.047). There was no difference in mean cortisol levels between SCORAD groups (*p* = 0.776). Morning basal serum cortisol values were observed above the upper limit of the reference range (171–536 nmol/L from 7 am to 10 am) in two (4%) patients in the AD group and one (2%) subject in the control group. A total of 14 (29%) AD and 4 (8%) control patients had lower values according to the reference range. The other 36 (69.23%) patients in the AD group and 50 (90.9%) subjects in the control group had normal levels of serum cortisol.

Table 2 shows the mean CBCL 6/18 and T-scores for AD patients and controls. The results of the total behavior problem showed that only one (2%) subject in the AD group and two (4%) subjects in the control group had scored above 70, corresponding to the clinical syndrome. A borderline disorder can be identified in one (2%) of the control group patients. The other 47 (98%) AD and 50 (97%) control patients had a total score below 64.

Differences in the overall scores of the three scales between the control and AD groups were found. AD patients had higher internal behavior scale scores and T-scores (6.6 ± 6.4 vs. 9.6 ± 6.9 and 47.9 ± 9.5 vs. 52.3 ± 10.2, *p* = 0.01), anxiety/depression scale score and T-score (2.8 ± 2.7 vs. 4.3 ± 3.5 and 47.7 ± 8.4 vs. 52.5 ± 11, *p* = 0.02), and somatic problems scale score and T-score (2.1 ± 2.3 vs. 3.5 ± 3 and 47.6 ± 8.5 vs. 52.7 ± 10.9, *p* = 0.005).

The mean total behavior problem score and the mean T-score were higher in the AD group than in the control group (26.1 ± 16.3 vs. 22.2 ± 17.3 and 51.2 ± 9.7 vs. 48.9 ± 10.2), but this difference was not statistically significant (*p* = 0.134). However, when the data were stratified by sex and age (6–11 and 12–18 years), there was a statistically significant difference between the groups of girls aged 6–11 years. Girls with AD had a higher total behavior problem scale score than girls in the control group (31.8 ± 19.37 vs. 22.35 ± 7.29, *p* = 0.036). We also found that in this age (6–11 years) sample, the scores on the social difficulties and thought problem scale for AD girls were higher than those of the control group. The mean score for the social difficulties scale in AD groups was 3.13 ± 2.80 vs. control 1.41 ± 1.22 (*p* = 0.014) and in thought problem scales, respectively, 2.8 ± 2.31 vs. 1.35 ± 0.97 (*p* = 0.013).

In the groups of boys aged 6–11 years and 12–18 years, differences were found only on the somatic problems scale scores; boys with AD had higher results than boys from control groups. The results are ordered as follows: 2.8 ± 2.31 vs. 1.35 ± 0.97 (*p* = 0.013) in the 6–11 years groups and 5.2 ± 4.53 vs. 2 ± 2.73 (*p* = 0.03) in the 12–18 years groups. The other clinical range of the CBCL 6/18 scores was not different between the groups. A summary of the results of the CBCL 6/18 questionnaire comparing the data of children with AD and healthy controls is in Table 3.

We compared the CBCL scale scores and T-scores between different AD severity groups (Table 4). A total of 19 (40%) patients had severe AD and 29 (60%) had mild-to-moderate AD. Patients with severe AD experienced more sleep disturbances and a greater intensity of itching. Their sleep disturbance and itching scores were higher than those of patients with mild–moderate AD (5.4 ± 2.6 vs. 2.4 ± 2.2, *p* = 0.000 and 6.6 ± 2.4 vs. 4 ± 2.8, *p* = 0.001). However, the total behavior problem score and other clinical ranges of the CBCL scores were not significantly different between the severe and mild-to-moderate AD groups.

To test which factors have the highest influence, we tried to model behavioral scales by factors—severity by SCORAD, age, sex, morning cortisol level, the duration of disease, the intensity of pruritus, and the intensity of sleep disturbance. Below we provide the summary in Table 5, which includes all factors and, as can be seen, none of them are statistically significant.

## 4. Discussion

The results of our study revealed that patients with AD (aged 6–18 years) have higher mean scores in total behavior problems, internalizing behavior (anxiety/depression, somatic complaints), and social and thought problems scales compared to controls. Depression and anxiety are common mental disorders found in patients with chronic inflammatory diseases [10]. Depression and anxiety are highly prevalent psychiatric disorders in children and adults with AD [10,11,12,15,16,17]. Moraes et al. [17] observed that almost half of the AD patients in their study had CBCL internalizing problem values within the clinical range. Muzzolon et al. [16] revealed that children and adolescents with AD present a higher risk for internalizing disorders in comparison with healthy siblings. These findings are supported by evidence in the literature suggesting that AD may contribute to the development of BDs [11,13].

People with severe AD usually have more frequent exacerbations and more severe symptoms. One of the hypotheses is that more severe AD may have a greater impact on patients’ mental health than milder AD. Park et al. [15] revealed that children and adolescents with severe AD more frequently have internalizing problems, particularly isolation (withdrawal/depression), somatic complaints, and anxiety problems. Although we found differences between AD patients and healthy controls, the results of our CBCL questionnaire were not significantly different when comparing severe and mild–moderate AD groups.

Our results show that patients with severe AD have more sleep disturbances and more itching scores compared to mild-to-moderate AD patients. An inadequate quantity and quality of sleep are associated with various impairments in daytime performances and can impair aspects of emotional and cognitive functioning [7]. Camfferman et al. [6] found that children with eczema had a significantly higher number of scratching events that originated during wake and sleep and a longer duration of scratching events during sleep, than controls. In a study by Ramirez et al. [5], children with active atopic dermatitis had poorer sleep quality, and the association was strongest among children with more severe disease. These findings are consistent with Fishbein et al. [24], a cross-sectional study of clinic populations that used objective measures of sleep, including actigraphy and polysomnography. The authors found that children with moderate-to-severe AD experience lower sleep efficiency than healthy controls. Similar results were shown by Moraes et al. [17]; they observed a higher prevalence of sleep disturbance in patients with AD. In comparing the mild and moderate–severe AD groups, Moraes et al. - [17] observed a higher prevalence of sleep difficulties and aggressive behaviors in patients with moderate–severe AD.

In contrast with Moraes et al., rates of aggressive behaviors scales in our study were not different between the severe and mild-to-moderate AD group and AD and control patients’ groups. Aggressive behavior varies according to age and gender and may also be influenced by the culture and customs of a family or country.

We observed that boys with AD have higher somatic complaints and girls higher social and thought problems scores in comparison with control. The good control of the disease requires a consistent skin care and treatment routine, which is applied from infancy. Deviations from the routine almost always lead to the worsening of the disease, as well as the worsening of the symptoms. This is an assumption, but a possible explanation could be that, at least in our country, women are more likely to take care of their skin, whereas for girls, social connection and communication are very important.

Children and adolescents, whether healthy or chronically ill, have the same needs during development. Nevertheless, it is more difficult for a sick child to meet the needs specific to each developmental stage of the child and at the same time to cope with the stressors of chronic illness. The symptoms and conversion of the illness can alter the child’s and adolescent’s mental development, as well as interfere with their interaction with their environment [17].

Based on previous studies, the psychological stress caused by AD itself seems to trigger immunological problems and worsen AD symptoms, creating a vicious cycle that can lead to sleep difficulties and psychological and behavioral problems [10,16,25,26]. In addition, hormonal disruption found in AD patients can directly cause psychological and behavioral problems [27]. We found that the mean cortisol level was statistically lower in the AD group compared to the healthy control group. The hypothalamic–pituitary–adrenal (HPA) axis represents a major immunoregulatory system that plays an important role in balancing the immune response, especially under stressful conditions. Stress-induced immunomodulation is altered in patients with AD, but the exact mechanisms are not well understood. Recent studies have shown that in patients with AD, for example, psychological stress can impair or blunt the HPA axis reactivity, and it can over-activate the sympathetic system, which may lead to an increased Th2 response and aggravation of the symptoms. Additionally, AD can lead to psychological stress, due to stigmatization, social isolation, and discrimination [28]. Several studies have looked at cortisol levels “at rest” in patients with allergic disorders versus nonatopic control patients, and the results are variable. A study by Kojima et al. [29] revealed that salivary cortisol responsiveness to the stress of venipuncture is correlated negatively with the severity of AD. In the study by Afsar et al. [27], data analysis showed no statistical difference in the basal serum cortisol levels (*p* = 0.383) between the AD and healthy controls, and the SCORAD index did not correlate with the basal serum cortisol values in AD patients (*p* = 0.06). Tehranchinia et al. [30] observed no statistical difference between the AD patients and healthy controls for basal serum cortisol and ACTH levels. Several studies found a relationship between cortisol levels and the severity of disease. Vinnik et al. [31] showed that all patient serum cortisol levels were within normal ranges; however, AD male cortisol was higher, and cortisol levels were found to be negatively correlated with SCORAD. Nutan et al. [32] showed that half the patients with AD had low basal cortisol levels and this is more marked in patients with severe AD. The populations and methodologies of the studies that we reviewed during the literature analysis differed, making it difficult to generalize the data. Several studies, like ours, showed that although mean cortisol values were within the normal range [27,30,31,33,34], AD patients had a significantly lower mean value and a higher number of patients had lower than normal serum cortisol levels compared to healthy controls [28,33]. Our study, like others, did not find significant changes in mean cortisol levels concerning disease severity [27], although some authors have found this relationship [31,32].

In our study, cortisol levels were not an associated risk factor for behavioral problems, and we found only one study that looked at the relationship between cortisol levels and behavioral problems. Afsar et al. [27] did not find a significant correlation between the morning basal cortisol values and anxiety levels in children with AD (the scores were obtained from the State-Trait Anxiety Inventory for Children (STAI-C), whereas there was a correlation between the morning basal serum cortisol values and the scores of the TAI-C in the control group.

Disease duration, but not clinical severity such as SCORAD, was an independent risk factor for having internalizing problems in AD children, in the study by Park et al. [15]. This suggests that patients experienced the psychosocial burden associated with a chronic, persistent course of illness. Our study did not support this hypothesis. None of the tested factors (severity by SCORAD, age, sex, morning cortisol level, the duration of disease, the intensity of pruritus, and the intensity of sleep disturbance) were statistically significant and did not have associations with behavioral problems in our analysis.

In our opinion, the difference in the results shows that behavioral difficulties can be the result of a variety of factors, both physiological and individual, related to the disease or the living environment. Their identification requires a long-term and detailed assessment of the patient’s history and clinical, physiological, and psychological features.

Our study had some limitations in identifying the links between AD and behavioral difficulties; overall, it can be described as a small sample study, and it could benefit from broader psychological research/exploration, such as cognitive function and stigma assessment. Furthermore, data on the behavior of the participants were only obtained from a single parent accompanying the patient. Longitudinal studies may verify the persistence of symptoms, as well as the conditions associated with their attenuation or intensification over time.

## 5. Conclusions

Children with AD (from 6 to 18 years) have higher internal behavior, anxiety/depression, and somatic problems scale scores and T-scores compared with healthy controls. Girls with AD between 6 and 11 years old have a higher total behavior problem score, social difficulties, and thought problem scales scores compared with healthy 6–11-year-old girls, whereas boys with AD between 6 and 11 years old and 12 to 18 years old have higher somatic complaints scale scores compared with healthy controls.

Patients with severe AD experience more sleep disturbances and a greater intensity of itching compared to mild–moderate AD. In our study population, the AD severity measured by SCORAD, age, gender, morning cortisol level, the duration of illness, the intensity of pruritus, and the intensity of sleep disturbance did not have a statistically significant effect on the development of behavioral problems. Recognizing mental disorders in individuals with AD and achieving effective treatments for mental symptoms might significantly improve patients’ health and quality of life.

## Figures and Tables

**Table 1:** The demographic, clinical characteristics, and cortisol levels of the participants.

Variable	Value Control Group	Value AD Group	Value AD Mild–Moderate Group (According to SCORAD)	Value AD Severe Group (According to SCORAD)
Total, *n* (%)	53 (52%)	48 (48%)	29 (60%)	19 (40%)
Age, years, mean ± SD	10.5 ± 3.1	10.5 ± 3.1	9.5 ± 2.6	10.8 ± 3.3
Sex girls/boys, *n* (%)	27 (49%)/ 26 (57%)	28 (51%)/ 20 (43%)	16 (55%)/ 13 (45%)	12 (63%)/ 7 (37%)
Family history of atopy, *n* (%)	24 (45%) *	32 (67%) *	-	-
Comorbidities Rhinitis Asthma Food allergy, *n* (%)	-	31 (65%) 25 (52%) 8 (21%) 17 (35%)	-	-
Duration of AD (months), mean ± SD	-	111.96 ± 34.78	105.7 ± 30.7	121.5 ± 39
SCORAD mean ± SD	-	41.75 ± 20.95	-	-
Sleep disturbance mean ± SD, points, *n* (%)	-	3.56 ± 2.77	2.4 ± 2.2 *	5.4 ± 2.6 *
Intensity of pruritus, points, mean ± SD	-	5.04 ± 2.9	4 ± 2.8 *	6.6 ± 2.4 *
Cortisol, mean ± SD, nmol/L	351.55 ± 126.1 *	252.9 ± 304.34*	257.3 ± 115.1	269.4 ± 119.1

Abbreviations: AD—atopic dermatitis, SCORAD—SCORing AD index, SD—standard deviation, * *p*-value < 0.05.

**Table 2:** Results of the CBCL 6/18 scores and T-scores in children with AD and controls.

Syndromes	Group	Mean ± SD [Median (Q1–Q3)]	T-Score Mean ± SD [Median (Q1–Q3)]	*p*-Value
Internalizing behavior scale	Control	6.6 ± 6.4 [5.0 (3–8)]	47.9 ± 9.5 [45.6 (42.7–50.5)]	0.010
Internalizing behavior scale	AD	9.6 ± 6.9 [8.5 (4–13)]	52.3 ± 10.2 [50.7 (44.1–57.3)]	0.010
Anxiety/Depression scale	Control	2.8 ± 2.7 [2.0 (1–4.1]	47.7 ± 8.4 [45.3 (42.1–51.6)]	0.020
Anxiety/Depression scale	AD	4.3 ± 3.5 [3 (2–6.3)]	52.5 ± 11 [48.4 (45.3–58.6)]	0.020
Isolation (Withdrawn/Depression scale)	Control	1.7 ± 2.5 [1 (0–3)]	49.9 ± 10.9 [46.8 (42.4–55.5)]	0.564
Isolation (Withdrawn/Depression scale)	AD	1.8 ± 2.1 [1 (0–3)]	50.1 ± 9 [46.8 (42.4–55.5)]	0.564
Somatic problem scale	Control	2.1 ± 2.3 [2 (0–3)]	47.6 ± 8.5 [47.3 (40–50.9)]	0.005
Somatic problem scale	AD	3.5 ± 3 [3 (1.8–5)]	52.7 ± 10.9 [50.9 (46.4–58.2)]	0.005
Externalizing behavior scale	Control	5.8 ± 4.6 [5 (2–9)]	49.6 ± 9.3 [48.1 (42–56.2)]	0.819
Externalizing behavior scale	AD	6.1 ± 5.3 [5.0 (2.0–9.3)]	50.4 ± 10.8 [48.1 (42.0–56.7)]	0.819
Rule-breaking behavior scale	Control	1.9 ± 2.0 [1 (0–3)]	50.9 ± 10.7 [46.0 (40.6–56.7)]	0.433
Rule-breaking behavior scale	AD	1.6 ± 1.7 [1 (0–2.3)]	49.0 ± 9.2 [46.0 (40.6–52.7)]	0.433
Aggressive behavior	Control	3.8 ± 3.3 [3 (1–6)]	49.0 ± 8.7 [46.8 (41.5–54.8)]	0.520
Aggressive behavior	AD	4.6 ± 4.2 [3.5 (1–7)]	51 ± 11.2 [48.2 (41.5–57.5)]	0.520
Social problem scale	Control	1.9 ± 2.2 [1 (0–3)]	48.8 ± 10.1 [44.7 (40.2–53.8)]	0.104
Social problem scale	AD	2.5 ± 2.2 [2 (1–4)]	51.3 ± 9.8 [49.2 (44.7–58.3)]	0.104
Thought problem scale	Control	1.9 ± 2.4 [1 (0–3)]	49.2 ± 10.1 [45.3 (41–53.9)]	0.279
Thought problem scale	AD	2.3 ± 2.3 [2 (0–3.3)]	50.9 ± 9.9 [49.6 (41.0–54.9)]	0.279
Attention problems	Control	3.5 ± 3.4 [3 (1–5)]	50.2 ± 11.1 [48.7 (42.2–55.2)]	0.856
Attention problems	AD	3.3 ± 2.7 [3 (1.0–4.3)]	49.7 ± 8.7 [48.7 (42.2–52.8)]	0.856
Total behavior problem scale	Control	22.2 ± 17.3 [22 (11–26)]	48.9 ± 10.2 [48.8 (42.3–51.2)]	0.134
Total behavior problem scale	AD	26.1 ± 16.3 [25.5 (12.5–37.5)]	51.2 ± 9.7 [50.9 (43.2–58)]	0.134

Abbreviations: AD—AD, CBCL 6/18—2001 Child Behavior Checklist for Ages 6–18, SD—standard deviation.

**Table 3:** Summary of the results of the AD children’s CBCL 6/18 questionnaire, by gender (boys and girls) and age group (6–11 and 12–18).

Syndromes	Results of an AD Group
Internalizing problems	↑ AD 6–11 years ↑ AD 12–18 years
Anxiety/Depression	↑ AD 6–11 years ↑ AD 12–18 years
Isolation (Withdrawn/Depression scale)	-
Somatic Complaints	↑ AD boys 6–11 years ↑ AD boys 12–18 years ↑ AD 6–18 years
Externalizing problems	-
Rule-Breaking Behavior	-
Aggressive Behavior	
Social Problems	↑ AD girls 6–11 years
Thought Problems	↑ AD girls 6–11 years
Attention Problems	-
Total behavior problem score	↑ AD girls 6–11 years

Abbreviations: AD—atopic dermatitis, CBCL 6/18—Child Behavior Checklist for Ages 6–18.

**Table 4:** Results of the CBCL scores and T-scores in children with severe and mild-to-moderate AD.

Syndromes	AD Severity According to SCORAD	Mean ± SD [Median (Q1–Q3)]	T-Score Mean ± SD [Median (Q1–Q3)]	*p*-Value
Internalizing behavior scale	severe	9.6 ± 7.0 [11.0 (4–13.5)]	52.4 ± 10.3 [54.4 (44.1–58.1)]	0.841
Internalizing behavior scale	mild–moderate	9.5 ± 7 [8 (4–12)]	52.2 ± 10.3 [50 (44.1–55.9)]	0.841
Anxiety/Depression scale	severe	4.7 ± 3.9 [3 (1.5–7.5)]	53.9 ± 12.3 [48.4 (43.7–62.6)]	0.648
Anxiety/Depression scale	mild–moderate	4.0 ± 3.2 [3 (2–5)]	51.7 ± 10.2 [48.4 (45.3–54.7)]	0.648
Withdrawn/Depression scale	severe	1.4 ± 2.4 [0 (0–2)]	48.6 ± 10.6 [42.4 (42.4–51.1)]	0.095
Withdrawn/Depression scale	mild–moderate	2.0 ± 1.8 [2 (0–4)]	51.1 ± 7.9 [51.1 (42.4–59.8)]	0.095
Somatic problems scale	severe	3.5 ± 2.9 [3 (1.5–5)]	52.7 ± 10.5 [50.9 (45.5–58.2)]	0.932
Somatic problems scale	mild–moderate	3.5 ± 3.1 [3 (2–5)]	52.7 ± 11.4 [50.9 (47.3–58.2)]	0.932
Externalizing behavior scale	severe	6.8 ± 6.9 [5 (2–11)]	51.7 ± 13.9 [48.1 (42–60.3)]	0.949
Externalizing behavior scale	mild–moderate	5.7 ± 4.1 [5 (2–9)]	49.6 ± 8.3 [48.1 (42.0–56.2)]	0.949
Rule-breaking behavior scale	severe	1.5 ± 2.2 [1 (0–2)]	48.5 ± 11.7 [46.0 (40.6–51.3)]	0.252
Rule-breaking behavior scale	mild–moderate	1.6 ± 1.4 [1 (1–3)]	49.3 ± 7.3 [46 (46–56.7)]	0.252
Aggressive behavior scale	severe	5.3 ± 5.1 [3 (2–8.5)]	53 ± 13.6 (46.8 [44.2–61.4])	0.497
Aggressive behavior scale	mild–moderate	4.1 ± 3.6 [4 (1–7)]	49.8 ± 9.5 [49.5 (41.5–57.5)]	0.497
Social problem scale	severe	2.5 ± 2.3 [2 (1–3)]	51.4 ± 10.4 [49.2 (44.7–53.8)]	0.949
Social problem scale	mild–moderate	2.4 ± 2.1 [2 (1–4)]	51.3 ± 9.6 [49.2 (44.7–58.3)]	0.949
Thought problem scale	severe	2.5 ± 2.2 [2 (0.5–4)]	51.8 ± 9.6 [49.6 (43.2–58.1)]	0.465
Thought problem scale	mild–moderate	2.2 ± 2.4 [2 (0–3)]	50.3 ± 10.2 [49.6 (41.0–53.9)]	0.465
Attention problems scale	severe	4.0 ± 2.9 [3 (2–6.5)]	52 ± 9.4 [48.7 (45.5–60.1)]	0.217
Attention problems scale	mild–moderate	2.9 ± 2.5 [3 (1–4)]	48.3 ± 8.1 [48.1 (42.2–52)]	0.217
Total behavior problem scale	severe	27.3 ± 10.3 [27 (11–40.5)]	51.9 ± 11.5 [51.8 (42.3–59.8)]	0.677
Total behavior problem scale	mild–moderate	25.3 ± 14.4 [25 (13–37)]	50.7 ± 8.5 [50.6 (43.5–57.7)]	0.677
Pruritus intensity score	severe	6.6 ± 2.4 [6 (5–8.5)]	5.4 ± 2.6 [5 (5–7.5)]	0.001
Pruritus intensity score	mild–moderate	4 ± 2.8 [3 (2–5)]	2.4 ± 2.2 [2 (1–4)]	0.001

Abbreviations: AD—atopic dermatitis, SCORAD—SCORing atopic dermatitis index, SD—standard deviation.

**Table 5:** Multivariate linear regression of factors influencing CBCL 6/18 behavioral scales.

Indicator	Driver	Coefficient	*p*_Value	r
Internalizing behavior scale	(Intercept)	18.861		0.108
Internalizing behavior scale	SCORAD	0.027	0.833	0.108
Internalizing behavior scale	Age	−0.162	0.922	0.108
Internalizing behavior scale	Sex—man	−1.173	0.702	0.108
Internalizing behavior scale	Morning cortisol level	−0.019	0.140	0.108
Internalizing behavior scale	Duration of disease (months)	−0.014	0.921	0.108
Internalizing behavior scale	Intensity of pruritus	−0.568	0.455	0.108
Internalizing behavior scale	Intensity of sleep disturbance	0.473	0.595	0.108
Externalizing behavior scale	(Intercept)	10.475		0.282
Externalizing behavior scale	SCORAD	0.164	0.087	0.282
Externalizing behavior scale	Age	−1.488	0.224	0.282
Externalizing behavior scale	Sex—man	3.019	0.184	0.282
Externalizing behavior scale	Morning cortisol level	−0.012	0.226	0.282
Externalizing behavior scale	Duration of disease (months)	0.075	0.455	0.282
Externalizing behavior scale	Intensity of pruritus	−0.182	0.743	0.282
Externalizing behavior scale	Intensity of sleep disturbance	−0.409	0.530	0.282
Total behavior problem scale	(Intercept)	50.415		0.212
Total behavior problem scale	SCORAD	0.314	0.271	0.212
Total behavior problem scale	Age	−2.696	0.464	0.212
Total behavior problem scale	Sex—man	3.361	0.621	0.212
Total behavior problem scale	Morning cortisol level	−0.053	0.069	0.212
Total behavior problem scale	Duration of disease (months)	0.085	0.779	0.212
Total behavior problem scale	Intensity of pruritus	−1.022	0.544	0.212
Total behavior problem scale	Intensity of sleep disturbance	−0.230	0.907	0.212
Indicator	Driver	Coefficient	*p*_value	r
Internalizing behavior scale	(Intercept)	18.861		0.108
Internalizing behavior scale	SCORAD	0.027	0.833	0.108
Internalizing behavior scale	Age	−0.162	0.922	0.108
Internalizing behavior scale	Sex—man	−1.173	0.702	0.108

Abbreviations: SCORAD—SCORing atopic dermatitis index.

## Data Availability

The raw data supporting the conclusions of this article will be made available by the authors on request.
